# Supplementary material for: Cardiac catheterization real-time dynamic radiation dose measurement to estimate lifetime attributable risk of cancer
Source: PLoS One. 2020 Jun 16;15(6):e0234461. doi: 10.1371/journal.pone.0234461 (PMC7297332; doi:10.1371/journal.pone.0234461)
Supplement: Attachment — Submitted filename: Response to Reviewers.docx [file pone.0234461.s003.docx]

Response to decision letter

Dear Editor, Dear reviewers

We thank the reviewers again for the helpful comments. The following is a point-by-point response to each comment:

Reviewer #2: The operator exposure still needs to be more detailed.

1. What specific assumptions have the authors make about LAR?

-a)-Constant yearly exposure from age 18 through 65? Probably not an entirely valid assumption since Cardiologist don't start at age 18, and probably don't do as many cases when starting, also have longer exposure per case when they are learning.

b) -- Do operators continue through age 65 or does the LAR assume that they have stopped at the time of the study?

c) is it possible to produce a survival analysis curve of the LAR?

**Author response:**

*For question a and b*

*Thank you for pointing this out. In our study, the LAR of all cancer incidence and mortality at the radiation dose for operators were calculated by BEIR VII Report. The BEIR VII report is based on a wide enough range of exposure to support meaningful statistical modeling. However, according to the LNT model adopted in BEIR VII, 10 mSv on a yearly basis from ages 18 to 65 years old, cancer incidence was 3,059 for male and 4,295 for female. That data was directly applied to the calculation of cancer risk to medical staff, so there is limitation for generalization in our results.*

*We have added more specific details in discussion as follows:*

*We have rephrased the statement in line 253-260 as “In addition, our study did not estimate the yearly of radiation exposure exactly and job tenure of operators. However, according to the BEIR VII report, when exposed continuously to 10 mSv on a yearly basis from ages 18 to 65 years old, cancer incidence was 3,059 for male and 4,295 for female. That data was directly applied to the calculation of cancer risk to medical staff in many studies [10, 58-60]. Although the LNT model for low-dose (<100mSv) is characterized by a great deal of uncertainty, there is no direct evidence to estimate the cancer risks for staff during the cardiac catheterization procedures.”*

*Newly added references:*

*58.	Cho H-O, Park H-S, Choi H-C, Cho Y-K, Yoon H-J, Kim H-S, et al. Radiation dose and cancer risk of cardiac electrophysiology procedures. International Journal of Arrhythmia. 2015;16(1):4-10.*

*59.	Mehta S. Health risks of low level radiation exposures: a review. Ind J Nucl Med. 2005;20:29-41.*

*60.	Kim JB, Lee J, Park K. Radiation hazards to vascular surgeon and scrub nurse in mobile fluoroscopy equipped hybrid vascular room. Annals of surgical treatment and research. 2017;92(3):156-63.*

*For question c*

*Thank you for your kind advice. However, a survival analysis curve requires prolonged observation and studies to rule out many potentially personal, mechanical, and environmental factors. We really appreciate your precious advice and useful suggestions for our future studies.*

2. Using a two month average is subject to determine case volume is subject to a good deal of error as there are seasonal variations in catheterization lab volume. Is it possible to use a 12 month average for the preceding year?

**Author response:**

*Thank you for being patient and helping me improve.*

*We have modified the case volume of 1 year occupational radiation exposure to the operators in abstract and results as follows:*

*We have rephrased the statement in line 32-35 (abstract) as LAR of all cancer incidences for staffs aged from 18 to 65 are varied from “0.40 %” for males to “1.50 %” for females. LAR of all cancer mortality for staffs aged from 18 to 65 are varied from “0.22 %” for males to “0.83 %” for females.*

*We have rephrased the statement in line 155-158 (results) as* *“We used a 12 month average for the preceding year to estimate the 1 year occupational radiation exposure to the operators is presented in Fig. 3. The annual average radiation dose per primary operator from all procedures was 3.49 mSv. As to the assistant operator was 1.30 mSv.”*

*We have rephrased the statement in line 160-162 (results) as LAR of all cancer incidence and mortality for male primary operators aged from 18 to 65 were “1.07 %”, and “0.59 %”, respectively. As to the female primary operators aged from 18 to 65 were “1.50 %”, and “0.83 %”, respectively.*

*We have modified the Table 2 as follows:*

**Table 2 Estimated LAR of all cancer incidence and mortality from cardiac catheterization procedures for operators**

| Operators | Primary operators | | Assistant operators | |
| --- | --- | --- | --- | --- |
| LAR | All cancer incidence aged from 18 to 65 | All cancer mortality aged from 18 to 65 | All cancer incidence aged from 18 to 65 | All cancer mortality aged from 18 to 65 |
| Male | *1.07 %* | *0.59%* | *0.40 %* | *0.22 %* |
| Female | *1.50 %* | *0.83 %* | *0.56 %* | *0.31 %* |

3. The quality y of Figure 1 need to be improved. It is very difficult to read the figures.

**Author response:**

*Thank you for the correction. We have modified the Figure 1:*
